# Genetic Predisposition to Infectious Disease

**DOI:** 10.7759/cureus.3210

**Published:** 2018-08-27

**Authors:** Nikolai Klebanov

**Affiliations:** 1 Dermatology, Massachusetts General Hospital, Boston, USA

**Keywords:** genetics, snp, malaria, norovirus, gwas, dengue, dengue, creutzfeldt-jakob disease (cjd), hepatitis c, hepatitis b

## Abstract

In contemporary medical practice, approaches to infectious disease management have been primarily rooted in a pathogen-centered model. However, host genetics also contribute significantly to infectious disease burden. The fast expansion of bioinformatics techniques and the popularization of the genome-wide association study (GWAS) in recent decades have allowed for rapid and affordable high-throughput genomic analyses. This review focuses on the host model of infectious disease with particular emphasis placed on the genetic variations underlying observed infectious disease predisposition. First, we introduce observational twin-twin concordance studies of diseases such as poliomyelitis, tuberculosis, and hepatitis which suggest the important role of host genetics. We review the well-established links between specific genetic alterations and predisposition to malaria (*P. falciparum* and *P. vivax*), Creutzfeldt-Jacob disease (CJD), human immunodeficiency virus (HIV), and Norwalk virus. Finally, we discuss the novel findings yielded by modern GWAS studies, which suggest the strong contribution of immunologic variation in the major histocompatibility complex (MHC) to host genetic infectious disease susceptibility. Future large-scale genomic studies hold promise in providing insights into immunology-pathogen links and may allow for the development of personalized genomic approaches to infectious disease prevention and treatment.

## Introduction and background

The pathogen or germ theory of infectious disease was not always as universally accepted as it is today. Hippocrates (c.460–370 BC) considered *phtisis*, a disease now known as tuberculosis, to be hereditary rather than infectious as he observed that it commonly clustered within families [1]. Nearly five hundred years later, Galen (129–210 CE) proposed that *phtisis* may have a contagious nature, and he warned against close contact with those afflicted with this disease. The pathogen-focused infectious disease theory truly took its strong hold in contemporary medicine in the late 19th century. Groundbreaking work by German physician and microbiologist Robert Koch led to the isolation of *Mycobacterium tuberculosis* as the causative tuberculosis pathogen, and thus medical microbiology became established as an independent discipline [2]. The germ infectious disease theory has led to unparalleled benefits for mankind in the development of highly effective antibiotics and antiviral and anti-parasitic medications.

Infectious diseases exert significant selective genetic pressure, and the genes that are involved in immune response are exquisitely diverse [3]. These observations suggest a strong role for host genetic variability in the susceptibility to exogenous pathogens. These genetic links have been observed grossly in twin-twin concordance studies. Specific genetic mutations and variations have been strongly implicated in the literature to confer susceptibility or resistance to diseases such as malaria (*P. falciparum* and *P. vivax*), Creutzfeldt-Jacob disease (CJD), human immunodeficiency virus (HIV), and Norwalk virus. Multiple novel genomic studies have demonstrated strong associations between polymorphisms in multiple histocompatibility complex (MHC) and human leukocyte antigen (HLA) genes with infectious disease susceptibility. Here, we provide an overview of these findings and discuss the future directions of this research and implications for clinical practice.

## Review

Concordance studies

Several studies focusing on infectious disease among monozygotic (MZ) and dizygotic (DZ) twin pairs have suggested a genetic contribution to disease burden. A twin-family study of poliomyelitis revealed that 36% of MZ twin pairs presented with paralytic poliomyelitis, compared to only 6% among DZ twins [4]. Tuberculosis concordance was likewise shown to be significantly higher among MZ than DZ pairs [5]. An analysis of hepatitis B among a Chinese population showed higher rates of seroconcordance between MZ and DZ pairs compared to non-twin sibling controls [6]. A landmark adoptee study in a Danish population reported in 1988 by Sørensen et al. reveals an interesting finding regarding the likely heritability of infectious disease predisposition [7]. The study authors followed 960 families where children were placed early in life with adopted parents. The risk of the adopted child dying from certain causes was evaluated in the context of a biologic or adoptive parent dying from the same cause before the age of 50. If a biologic parent died from infectious disease before age 50, the adopted child had 5.81 greater risk of also dying from infection. Meanwhile, the relative risk (RR) for the child if the adoptive parent died from infection was close to unity. The high RR of 5.81 was greater than the RR associated with cardiac, cerebrovascular, oncologic, and natural causes for the biologic parent. The results of this groundbreaking study suggest a strong host genetic component in infection predisposition, possibly stronger than for those diseases commonly believed to be highly heritable such as malignancy or cardiovascular disease.

The “big six” of infectious disease genetics

Six important genetic relationships with infectious disease susceptibility have been discovered and repeatedly validated in the literature, and thus constitute very strong findings [8]. These associations are summarized in Table 1.

**Table 1:** The “big six” genetic variants relevant to infectious disease

Year	Disease	Gene	Effect	Notes
1954	Plasmodium falciparum malaria	Hemoglobin subunit beta (HBB)	Protective	Increased red blood cell (RBC) turnover and abnormal shape à increased uptake by macrophages; decreased parasite growth
1976	Plasmodium vivax malaria	Duffy antigen receptor (DARC)	Protective	Duffy glycoprotein is an RBC surface receptor for P. vivax; abnormal or missing receptor leads to impaired parasite penetration
1991	Creutzfeldt-Jacob disease (CJD)	PRiON protein (PRNP)	Susceptible	Homozygous PRNP mutations predispose to CJD. 51% of the general population is PRNP heterozygous
1995	Plasmodium falciparum malaria	Band 3 anion transport protein (SLC4A1)	Protective	Southeast Asian ovalocytosis (SAO) is similar (but distinct from) hereditary elliptocytosis. Entry of P. falciparum is impaired.
1996	Human immunodeficiency virus 1 (HIV-1)	Chemokine receptor 5 (CCR5)	Protective	Altered CCR5 co-receptor (such as seen in CCR5Δ32 mutation) leads to impaired viral T-cell entry and slower progression to acquired immunodeficiency syndrome (AIDS)
2003	Norwalk virus (norovirus)	Fucosyltransferase 2 (FUT2)	Protective	FUT2 variants lead to non-secretor phenotype; gut cells do not express ligands necessary for binding of the GII.4 norovirus strain

Selective pressure in populations residing in areas with high prevalence of malaria has favored the sickle cell trait [9], and there is evidence that this genetic selection still continues in the present day [10]. Variation in the HBB gene has also been found to be associated with malaria protection by GWAS [11], and interestingly, has been associated with a significant host to vector *P. falciparum* transmission [12]. Another protective variant against *P. falciparum* malaria is the genetic condition termed Melanesian ovalocytosis (also known as Southeast Asian ovalocytosis or SAO). In SAO, there is a defect in the band 3 protein, an RBC membrane protein, which causes the band 3 protein to ankyrin protein bond to be stronger than normal. Multiple consequences result from this genetic change including greater RBC robustness, reduced anion exchange, intracellular ATP partial depletion, and a decrease in antigen expression [13]. Due to a combination of these forces, the entry of *P. falciparum* malaria into the RBC is impaired. SAO is maintained as a balanced polymorphism among human populations [14], and to highlight the genetic selective pressure for this condition, there is a 35% incidence of SAO in the north coast of Madang Province in Papua New Guinea, a geographic location where malaria is endemic [15].

The Duffy blood group genotype is another important host genetic contribution to malaria susceptibility, specifically to the* Plasmodium vivax* species. In 1975, Miller et al. studied the blood types of 11 black and six white volunteers who had been exposed to bites of *P. vivax*-infected mosquitoes. The study authors demonstrated that individuals with Duffy-blood-group-negative erythrocytes were resistant to parasite invasion [16]. They coined the Duffy group negative genotype as FyFy*.* This study led to the discovery that Duffy group antigens that are present on the RBC surface are important recognition sites and entry points for the* P. vivax *malaria parasite. The Duffy genotype is another important example of genetic selection as *P. vivax*, which is widespread throughout tropical and subtropical areas, is absent in West Africa where more than 95% of the population is Duffy negative [17].

Viral disease also presents with three important host genetic variants affecting disease susceptibility. Mutations in the PRNP (PRiON protein) gene, which are strikingly common at 51% among the normal population, are known to be a predisposing factor for sporadic Creutzfeld-Jakob disease [18]. The human immunodeficiency virus 1 (HIV-1) uses the CCR5 co-receptor on the surface of CD4+ T-lymphocytes to recognize and enter the T-cell. Thus, variants in the CCR5 gene such as the CCR5Δ32 mutation have been strongly linked to slower (acquired immunodeficiency syndrome) AIDS progression and protection against HIV infection. The CCR5 gene has been studied in vitro as a promising target for HIV treatment [19]. Finally, susceptibility to infections with norovirus has been shown to be affected by the FUT2 genotype. Cells lining the gastrointestinal tract in humans present A, B, or O blood group antigens on their surface. Fucosyltransferase 2, the protein product of FUT2, is an important mediator of this antigen presentation. Mutant FUT2 thus yields gut lining cells that do not present these antigens, and individuals with this phenotype are termed non-secretors. The non-secretor phenotype has been shown to be protective against norovirus infection specifically with the GII.4 strain [20]. The strain-specificity limits the clinical application of the knowledge of this genetic host factor, as in a case series of four patients who were affected by norovirus gastroenteritis, all four patients tested negative on a consumer genetic test. Three of these four individuals were infected with the GI.6 norovirus strain [21].

Genome-wide association study (GWAS) of infectious disease susceptibility

GWAS have been instrumental in the discovery of several important host genetic associations with infectious disease course. The methodology of GWAS allows for the simultaneous assay of over 500,000 single nucleotide polymorphisms (SNPs) across thousands of individuals. The statistical analysis of GWAS involves curating those SNPs that are significantly correlated with cases of disease with a p-value below 5x10-8, which is termed “genome-wide significance”, This stringent significance cutoff is important for controlling the number of false-positive hits given that hundreds of thousands or sometimes one to two million SNPs are interrogated at once (although alternative cutoffs to the standard 5x10-8 have been proposed in the literature [22-23]). The GWAS approach is particularly strong in its ability to provide for a systematic, global, and unbiased search for strong disease association, and GWAS has been instrumental in identifying many significantly associated variants of modest effect size [24].

Notable confirmatory and new findings yielded by GWAS include the significant association of common SNPs with leprosy, malaria, meningococcal disease, dengue, and chronic hepatitis B and C [24-25]. GWAS of leprosy [26-27] (caused by *Mycobacterium leprae*) revealed strong associations with HLA-DR and NOD2 which encode recognition sites for pathogen-associated motifs, and IL23R, RIP2K, and TNFSF15 which are involved in the downstream pathogen immune inflammatory response. With regards to malaria, GWAS have confirmed ABO blood-group association with *P. falciparum* infection (such as variation at the HBB gene locus), but no novel insights have emerged [11,28]. In meningococcal disease (referring to invasive *Neisseria meningitidis* brain or bloodstream infections), strong associations emerged between Factor H (CFH) and other CFH-related genes such as CFHR3 and CFHR1 [29]. The identification of this locus as significant confirms the known important interaction between *N. meningitidis* and the human complement cascade. Analysis of severe dengue infection manifesting as dengue shock with GWAS has identified strong associations at the MICB (located within the MHC complex) and PLCE1 loci [30]. These results suggest that infection with *Flaviviridae* family members may rely on the immune response arm constituted by MICB signaling via natural killer (NK) cells.

Regarding chronic hepatitis B virus (HBV), GWAS have revealed that HLA-DP variation strongly influences chronic HBV infection [31], supporting prior observations that antigen-presentation is an important interaction point between HBV infection and human host response.

The IL-28B association with hepatitis C warrants special mention. The IL-28B gene codes for the cytokine interferon lambda (IFN-lambda). Single nucleotide polymorphisms (SNPs) in the IL-28B locus have been strongly associated with response to IFN-alpha treatment in hepatitis C [32,33]. Race including African-American and Hispanic race has a significant effect-modulatory contribution to this association. Although this is a genetically-robust association which has been validated with GWAS study, in the context of contemporary direct-acting antiviral (DAA) therapy, the effects of differing IL-28B genotypes has shown very small impact. Given this small impact once appropriate DAA therapy is initiated, routine genotyping for IL-28B is not recommended in clinical practice.

Apart from these specific findings, GWAS has yielded mainly associations with many human leukocyte antigen (HLA) and multiple histocompatibility locus (MHC) genes to infectious disease [8,25]. Nearly half of loci identified by GWAS focused on infectious disease susceptibility are located within the broad MHC region of chromosome 6 [24]. These findings emphasize the undeniably important role of complex host immune response in the interaction with invading pathogens.

Summary of host genetic patterns contributing to infectious disease response

**Figure 1:**
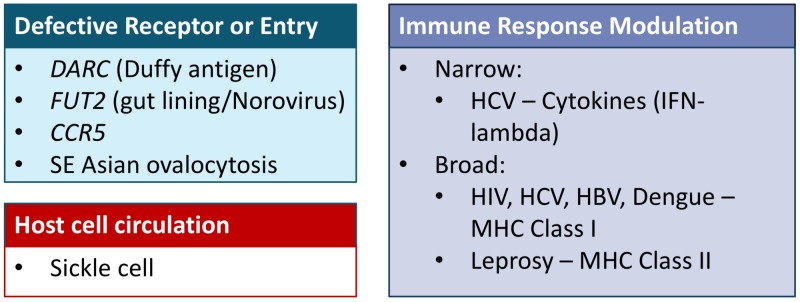
Summary of host genetic patterns contributing to infectious disease response

The main thematic groups of host genetic variations underlying disease susceptibility are summarized in Figure 1.

The relationships of DARC to *P. vivax*, FUT2 to norovirus, CCR5 to HIV-1, and SAO to *P. falciparum *constitute defective receptor or entry points which thus are protective against infection. This group of genetic variants is characterized by specific cell-surface protein alterations. Findings of such specific entry-point mutations are promising as possible therapeutic targets. The sickle cell anemia to *P. falciparum* malaria relationship falls under the rare category of alterations in the human host cell circulation patterns (specifically RBC longevity). Given the specific targeting of malaria to the RBC, this relationship is a unique one, and it is unlikely that other not yet discovered genetic variants would fall under this category. Finally, the category of immune response modulation captures the majority of the findings yielded by GWAS. There is the narrow immune system modulation such as the IL-28B or IFN-gamma relationship to chronic hepatitis C (HCV) infection. There is also a component of broad immune system variation such as the relation of multiple loci in the MHC region of chromosome 6 to many infectious disease entities.

## Conclusions

In this review, we have discussed the host-focused approach to infectious disease study (rather than the pathogen-focused one), with particular emphasis placed on specific human host genetic variations that predispose to changes in the response to invading pathogens. Candidate-gene studies throughout the past decades have led to the discovery and validation of well-established links between specific genetic alterations and predisposition to malaria (*P. falciparum* and *P. vivax*), Creutzfeldt-Jacob disease (CJD), human immunodeficiency virus (HIV), and Norwalk virus. Modern studies using the GWAS approach have yielded several novel findings and many confirmatory ones. Most GWAS-identified loci are located within the broad MHC region of chromosome 6, suggesting that infectious disease susceptibility is highly dependent across many pathogen entities to complex variation within the human immune response. These are interesting findings, but with only nascent robust ways to specifically target and modulate the immune system such as immunotherapy for malignancies, these findings are not currently clinically actionable. Future studies with in vitro and in vivo validation of these GWAS findings, and the expected further development of more highly-specific immunotherapies, are likely to yield promising results in the use of immune modulation for infectious disease treatment.
